# Commentary: Mending a broken heart: The use of durable mechanical circulatory support

**DOI:** 10.1016/j.xjtc.2021.03.008

**Published:** 2021-03-04

**Authors:** Francis D. Pagani

**Affiliations:** Department of Cardiac Surgery, University of Michigan, Ann Arbor, Mich

**Figure:**
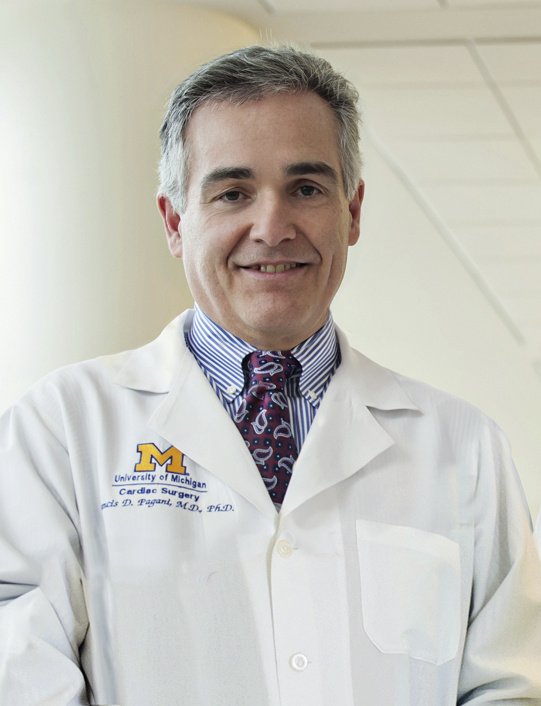
Francis D. Pagani, MD, PhD

**Central Message**

Myocardial recovery associated with durable mechanical circulatory support represents an important potential treatment paradigm but is poorly understood.

See Article page 182.

Durable left ventricular assist device (LVAD) therapy provides lifesaving benefit and improvement in quality of life for patients with advanced heart failure refractory to guideline-directed medical care. Although originally intended for bridge-to-transplant and later as permanent therapy (ie, destination therapy), mechanical unloading with durable LVADs has been associated with improvement in myocardial function that has permitted device explant with long-term freedom from heart failure in a small cohort of patients; that is, a bridge to recovery. This observation has raised the possibility that mechanical unloading with durable LVADs can facilitate myocardial recovery and alter the clinical phenotype of advanced heart failure.

Faerber and colleagues^1^ provide an expert review of the current state of using durable LVADs to promote myocardial recovery and achieve long-term freedom from heart failure. Faerber and colleagues^1^ discuss a number of important conceptual and practical issues currently surrounding the use of durable LVADs to promote myocardial recovery. First, although numerous studies have demonstrated improvement in the biology of the recovered heart associated with mechanical unloading, improvement in biology alone is insufficient to predict the degree of functional recovery or long-term freedom from heart failure. The use of the term *heart failure remission* rather than *recovery* reflects our uncertainty and the lack of a basic understanding of the recovery phenomenon. Second, the prevalence of myocardial recovery during support is generally believed to be low, but some important clinical characteristics such as young age, short duration of heart failure, nonischemic etiology of the heart failure, and smaller left ventricular volumes predict a higher prevalence of myocardial recovery. Aside from these factors, the ability to accurately predict with high probability those patients who might respond to mechanical unloading is lacking. Third, concomitant treatment with optimal medical therapy and use of guideline-directed heart failure therapies in addition to mechanical unloading is likely essential to the recovery paradigm. Fourth, we lack an understanding of how LVADs should be managed to promote myocardial recovery. Current LVADs are operated in a fix speed mode and unload the left ventricle during all phases of the cardiac cycle, conditions that may not optimize myocardial recovery. Further, the functional assessment of myocardial recovery and selection of patients for device explantation remains arbitrary, although some clinical weaning characteristics or criteria appear relevant to patient selection. Last, current device technology presents a formidable challenge to device removal in the setting of myocardial recovery. The complexity of device removal has some surgeons advocating for device decommissioning rather than device explantation. Whether device decommissioning and retention of the device in a young patient (most commonly the cohort likely to experience recovery), is safe over the long-term is not known.

Faerber and colleagues provide a succinct summary of the state of using durable LVADs to promote myocardial recovery. This is an important area that needs continued investigation to bring to reality a potentially important treatment paradigm and option for patients with advanced heart failure.
